# Bladder management for adults with spinal cord injury in the acute hospital setting: A retrospective study

**DOI:** 10.1038/s41394-026-00730-8

**Published:** 2026-03-05

**Authors:** Emily Hon, Mengdong He, Lin Xu, Stephen Hampton, Kimberly Waddell

**Affiliations:** 1 Department of Physical Medicine and Rehabilitation, University of Pennsylvania, Philadelphia, PA USA; 2 David Geffen School of Medicine at the University of California, Los Angeles, Los Angeles, CA USA; 3 Division of General Internal Medicine, Perelman School of Medicine, University of Pennsylvania, Philadelphia, PA USA; 4 Corporal Michael J. Crescenz VA Medical Center, Philadelphia, PA USA

**Keywords:** Rehabilitation, Epidemiology

## Abstract

**Study Design:**

Retrospective cohort study.

**Objectives:**

The purpose of this study was to describe bladder management during acute hospitalization for people with SCI and its association with length of hospitalization.

**Setting:**

Acute hospitals within a single academic health system in the United States of America.

**Methods:**

Data were extracted from the electronic health record for admissions between September 1, 2021, and September 30, 2023. Bladder management for all admissions was classified as either indwelling urinary catheter (IUC), clean intermittent catheterization (CIC), IUC and CIC, or no catheter. The relationship between bladder management, injury type, and length of stay was examined using a mixed effects linear regression model.

**Results:**

The final sample included 1169 unique patients and 1652 admissions. Half (49.7%) of admissions required no urinary catheter. The IUC-only group comprised 18.9% of admissions while CIC and the combined IUC and CIC groups comprised 15.7% of admissions. Most admissions (84.3%) included a single bladder management strategy. Up to 63.1% of the combined IUC and CIC group implemented a trial of CIC more than once during the admission. Both IUC and CIC management was associated with a significant increase in hospital stay of 10.7 days (95% CI [5.3, 16.1], P < 0.001) for those with a cervical injury.

**Conclusion:**

These results provide valuable information about existing care patterns that can guide future quality improvement initiatives to enhance bladder management early after SCI.

## Introduction

In the United States, approximately 296,000 individuals are living with a spinal cord injury (SCI), with 17,900 new cases of SCI annually [1]. Approximately 80% of individuals with chronic SCI are unable to void volitionally [2]. Bladder control is a complex process that regulated by the nervous system. As the bladder fills, sensory information is relayed from the bladder to the brain indicating a need to empty and the brain, in turn, sends activation signals to the bladder to coordinate emptying [3]. These signals travel through the spinal cord. A disruption to the nervous system – such as a SCI -- can disrupt these sensory signals and impair bladder control, which is called neurogenic bladder. Monitoring and managing bladder volumes in individuals with both acute and chronic SCI is a cornerstone of care that is addressed during hospitalization and continues throughout the disease course.

Failure to adequately manage bladder volumes in the SCI population can result in adverse health outcomes, such as autonomic dysreflexia (AD) or renal damage [4, 5]. This tends to be an insidious process, with bladder management influencing changes in bladder physiology over time [6]. Bladder overdistension, which is a state where the bladder exceeds its normal volume (typically ≥500 milliliters) [7], has been estimated to be responsible for up to 80% of AD cases in susceptible individuals, which is a life-threatening phenomenon [8]. Bladder overdistension increases intravesical pressure which can result in urine flowing backwards into the ureters and kidneys, causing renal damage. Maintaining appropriate bladder volumes, starting in the acute period following SCI, can help prevent these complications.

The indwelling urinary catheter (IUC) is a common method of bladder management in the acute hospital. Preference for IUC, however, is often low given its strong association with developing a UTI [9]. The catheter circumvents the protection offered by the urethral sphincter, acts as a nidus for infection, and can introduce trauma to the epithelium of the urinary tract, to which bacteria can then adhere [10]. Because catheter-associated UTIs (CAUTIs) are associated with significant increases in mortality, length of intensive care unit (ICU) and hospital admissions [11], and healthcare costs [12], national policies have been implemented to improve CAUTI prevention. First, the Agency for Healthcare Research and Quality introduced the national Comprehensive Unit-based Safety Program to reduce CAUTI in hospitals [13]. Second, the Centers for Medicare & Medicaid Services identified CAUTI as one condition within their hospital-acquired condition reduction program, where overall performance is tied to reimbursement [14]. Lastly, although not specific to SCI, clinical practice guidelines from the Infectious Diseases Society of America recommend IUC removal as soon as it is deemed feasible [15]. These national efforts have resulted in health systems making a conscious effort to discontinue IUC use early during hospitalizations, but it remains unclear how they have impacted care for populations with impaired bladder function, such as those with SCI.

Bladder management in the SCI population is often a complex decision that includes patient (age, emotional readiness) and clinical factors (level of injury, preserved hand function). This decision is also influenced by time. A spinal cord injury is a complex condition that undergoes dynamic changes over time, meaning that individual bladder management may need to be adjusted to account for functional gains or losses. For some individuals, such as those with tetraplegia or significant medical complexity, IUC may be the most appropriate bladder management strategy. For individuals who are clinically appropriate, the Consortium for Spinal Cord Medicine recommends clean intermittent catheterization (CIC) as the preferred method of bladder management for SCI-associated neurogenic bladder [16]. CIC allows the bladder to mimic the typical filling and emptying cycle, without the continual presence of a foreign instrument [4]. The extent to which CIC is initiated and sustained during acute hospitalization is unknown.

Understanding the types and duration of SCI bladder management – and their relationship to length of stay – during acute hospitalization is an important first step in understanding care patterns that can have a significant impact on health outcomes in this population. The purpose of this study was to quantify bladder management during acute hospital admissions, including the type and duration, and explore its association with hospital length of stay.

## Methods

This was a retrospective cohort study of individuals with a SCI who were admitted to a large, urban health system. Patients were included in this cohort study if they had a discharge diagnosis of SCI, identified by using ICD-10 diagnostic codes (Supplemental Table S1).

Additional study inclusion criteria were (1) acute hospital admission between September 1, 2021 and September 30, 2023, (2) length of admission ≥24 hours, (3) age ≥18 years, and (4) at least one documented bladder output during the acute admission. Patients were excluded if they died during hospitalization. This study was approved by the University of Pennsylvania Institutional Review Board and a waiver of informed consent was granted.

### Data sources and extraction

Patient-level data were extracted from the electronic health record (EHR) via Epic Clarity. Patient demographic variables included age, gender, and insurance type. Clinical variables included level of SCI (cervical versus thoracolumbar) and duration of catheter usage. In addition, bladder management orders and flowsheet data that documented the bladder output volume and the corresponding method (e.g., IUC) were extracted for each admission.

### Bladder management methods

All admissions were classified by the bladder management method into the following groups: IUC-only, CIC-only, IUC and CIC, or no catheter use. We identified the bladder management method for each urine output using the urine output measurement label from the EHR-extracted flowsheet data. For example, the EHR flowsheet label “R intermittent/straight cath (ml)” was classified as urine output obtained by CIC. For cases where measurements were recorded in the flowsheet under the nonspecific label “urine output,” we consulted the bladder management order file to determine if an order was active at the time of the recorded measurement. For example, if a bladder output volume was recorded under the label “urine output” and there was an active order at that time for maintenance of IUC, then the patient was considered to have IUC for bladder management. If no bladder management order was active during this time, the urine output volume was attributed to voiding with no catheter use. A single IUC or CIC measurement, with no other output volumes, qualified the admission for the IUC-only and CIC-only groups, respectively.

### Outcomes

Outcomes included the type of bladder management method, duration of bladder management method, and length of hospital admission. Duration of IUC and CIC were estimated by the time between first and last urine output measurements of the same type. If a patient was recorded as having only a singular CIC or a singular IUC measurement during the whole admission, they were excluded from the calculation of duration of CIC and IUC bladder management, respectively. Additionally, as CIC is typically performed multiple times a day, we sought to characterize CIC management including sporadic CIC measurements in a way reflecting true clinical practice. To do this, we capped time between CIC measurements to 24 hours. A group of CIC measurements where each sequential CIC measurement was less than 24 hours from the previous measurement, was called a “CIC stretch.” If the time between two consecutive CIC measurements exceeded 24 hours, then the latter CIC measurement was considered a new first CIC urine output in a separate CIC stretch. As a result, a single admission may have had multiple CIC stretches and, therefore, multiple CIC durations calculated. Length of admission was calculated as the duration, in days, between the admission and discharge dates.

### Statistical analysis

Cohort demographics, bladder management type and duration of IUC/CIC were evaluated descriptively and presented as mean (SD) for normally distributed continuous variables and as median [IQR] for non-normally distributed variables. Outcomes were analyzed for the full sample and then stratified by level of injury (thoracolumbar versus cervical).

The relationship between bladder management strategy and length of hospital admission was evaluated using a linear mixed effects regression model. The model included a patient random effect and adjusted for prior hospitalization for SCI (yes/no). Model covariates included age, sex, race, level of injury (cervical vs. thoracolumbar), and bladder management group. To understand the relationship between hospital length of stay, level of injury, and bladder management group, the model also included an interaction term for level of injury x bladder management group. All data analyses were performed with SAS statistical software version 9.4 with a *P* < 0.05 level of significance.

## Results

**Fig. 1:**
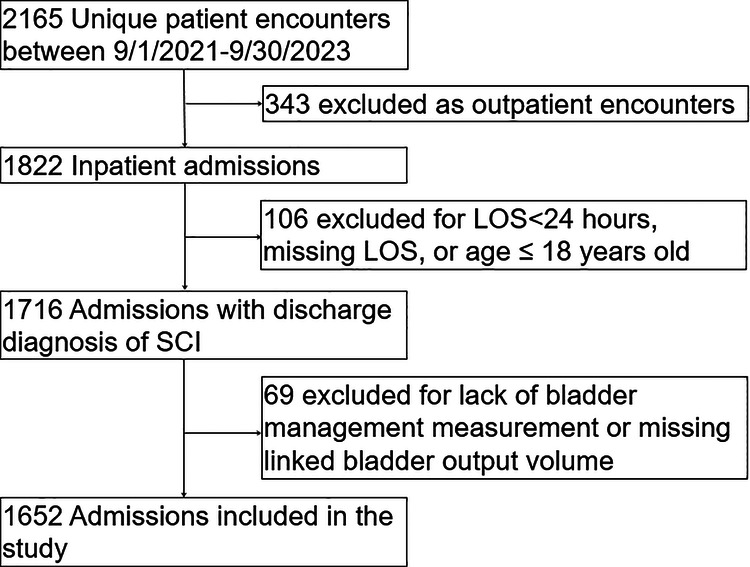
Flow diagram of cohort construction.

The final sample included 1169 unique patients and 1652 unique admissions (Fig. 1). The cohort was predominantly male (60.7%) with a mean (SD) age of 57.3 (19.3) years (Table 1). Most admissions were identified as either Medicare (56.7%) or Medicaid (25.4%) insurance type and a thoracolumbar SCI (76.3%). The sample was racially diverse, with 46.4% identifying as Black and 44.7% identifying as White.

**Table 1:** Baseline characteristics.

	Overall (n = 1652)	IUC only (n = 312)	IUC + CIC (n = 260)	CIC only (n = 259)	No Catheter (n = 821)
**Sociodemographics**					
Age, mean (SD)	57.3 (19.3)	54.0 (17.8)	56.2 (20.3)	54.1 (20.9)	59.9 (18.7)
Gender, n (%)					
Male, n (%)	1002 (60.7)	188 (60.3)	172 (66.2)	167 (64.5)	475 (57.9)
Female, n (%)	650 (39.3)	124 (39.7)	88 (33.8)	92 (35.5)	346 (42.1)
Race, n (%)					
Asian or Pacific Islander	47 (2.9)	8 (2.6)	5 (1.9)	4 (1.5)	30 (3.7)
Black or African American	760 (46.0)	153 (49.0)	119 (45.8)	124 (47.9)	364 (44.3)
Caucasian	731 (44.3)	122 (39.1)	112 (43.1)	116 (44.8)	381 (46.4)
Other	114 (6.8)	29 (9.3)	24 (9.2)	15 (5.8)	46 (5.6)
Ethnicity, n (%)					
Hispanic or Latino	56 (3.4)	14 (4.5)	7 (2.6)	10 (3.9)	25 (3.1)
Non Hispanic or Latino	1585 (95.9)	297 (95.2)	250 (96.2)	248 (95.7)	790 (96.2)
Other	11 (0.7)	1 (0.3)	3 (1.2)	1 (0.4)	6 (0.7)
Insurance Type, n (%)					
Medicare	931 (56.4)	156 (50.0)	119 (45.8)	155 (59.9)	501 (61.0)
Medicaid	418 (25.3)	86 (27.6)	81 (31.1)	69 (26.6)	182 (22.2)
Private Insurance	135 (8.2)	32 (10.2)	28 (10.8)	16 (6.2)	59 (7.2)
Managed Care	159 (9.6)	35 (11.2)	32 (12.3)	19 (7.3)	73 (8.9)
Other	9 (0.5)	3 (1.0)	0 (0.0)	0 (0.0)	6 (0.7)
Level of Injury, n (%)					
Cervical	392 (23.7)	85 (27.2)	65 (25.0)	42 (16.2)	200 (24.4)
Thoracolumbar	1260 (76.3)	227 (72.8)	195 (75.0)	217 (83.8)	621 (75.6)

**Fig. 2:**
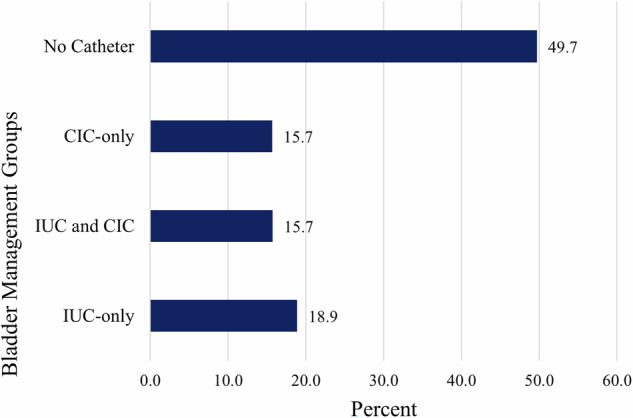
Proportion of Bladder Management Across All Admissions.

Nearly half (49.7%) of admissions required no urinary catheter to manage bladder needs. The next most common bladder management group was the IUC-only group, which encompassed 18.9% of the sample (Fig. 2). Lastly, 84.3% of all admissions included only a single bladder management method (including no catheterization) throughout the course of hospitalization.

After excluding admissions with only one IUC urine output, there were a total of 298 admissions used to calculate the duration (in days) of IUC bladder management in the IUC-only group. The median [IQR] duration of IUC bladder management in this group was 5.0 [3.0–10.0] days. A total of 250 admissions were used to calculate IUC duration in the combined IUC and CIC bladder management group. For this group, the median [IQR] duration of IUC bladder management was 9.0 [4.0–20.0] days.

There were 261 CIC stretches observed in the CIC-only group across 192 admissions, with a median [IQR] of CIC duration of 2.0 [1.0–4.0] days. There were a total of 305 CIC stretches observed in the combined IUC and CIC group, which were obtained from 187 admissions. The median [IQR] of CIC duration was 2.0 [1.0–5.0] days. Up to 63.1% of the combined IUC and CIC group implemented CIC more than once during an admission.

**Fig. 3:**
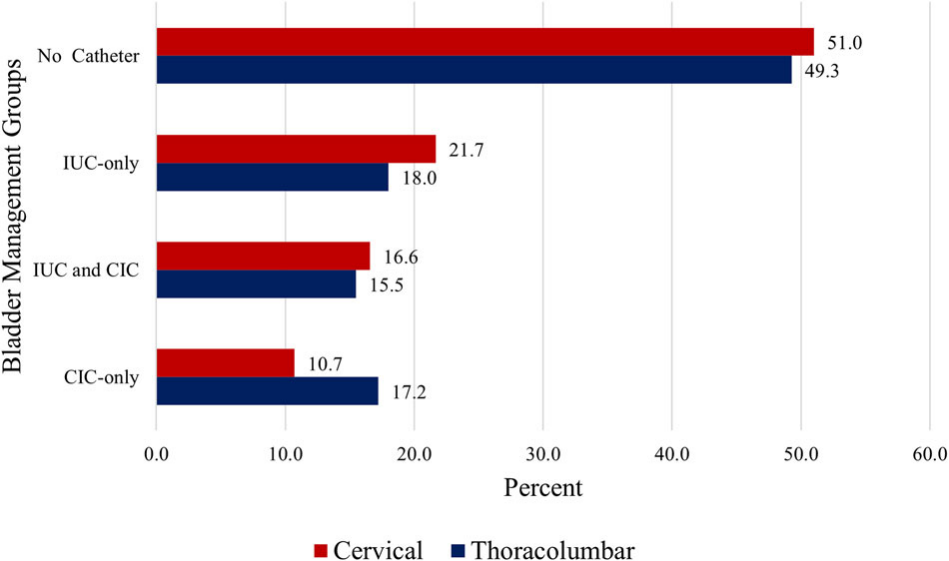
Proportion of Bladder Management Strategies by Level of Injury.

After stratifying the sample by SCI type (cervical versus thoracolumbar), approximately half of the cervical and thoracolumbar patients did not require a catheter for bladder management (51.0% cervical and 49.3% thoracolumbar). Patients with thoracolumbar injury comprised slightly more of the CIC-only group at 17.2%, compared to those with a cervical injury (10.7%) (Fig. 3). For those in the IUC-only group, the median [IQR] duration of IUC was 5.0 [3.0–10.0] days among those with a cervical injury and 5.0 [3.0–10.5] days for those with a thoracolumbar injury. In the combined IUC and CIC group, the median IUC duration for admissions with cervical SCI was 12.0 [4.0–29.0] days, compared to admissions with thoracolumbar SCI, which was 8.0 [4.0–16.5] days.

Results from the regression model demonstrated that, across all admissions, the mean length of stay was 8.6 days (Table 2). After adjusting for relevant covariates, we found a significantly increased length of stay associated with the IUC and CIC group (14.7 days, 95% CI [11.9, 17.4], *P* < 0.001) and IUC only group (3.1 days, 95% CI [0.5, 5.7], *P* = 0.01, Table 2). Within the IUC and CIC group, patients with a cervical injury – relative to those with a thoracolumbar injury – experienced a significantly longer hospital stay of 10.7 days (95% CI [5.3, 16.2), *P* < 0.001, Table 2).

**Table 2:** Adjusted Regression Results for Acute Hospital Length of Stay.

	Estimate (95% CI)	*P*
Intercept	8.6 (4.2, 13.0)	*<0.001*
Age	−0.1 (−0.1, −0.01)	*0.009*
Gender		
Female	Ref	*–*
Male	2.3 (0.4, 4.2)	*0.01*
Race		
White	Ref	*–*
Asian/Pacific Islander	−1.7 (−7.1, 3.5)	*0.51*
Black or African American	−0.4 (−2.5, 1.6)	*0.69*
Other	−0.4 (−5.5, 4.6)	*0.86*
Unknown	9.5 (4.5, 14.4)	*<0.001*
Cervical Injury	−0.1 (−2.9, 2.6)	*0.91*
Bladder Management Group		
No catheter use	Ref	*–*
CIC only	1.5 (−1.1, 4.1)	*0.26*
IUC & CIC	14.7 (11.9, 17.4)	*<0.001*
IUC	3.1 (0.5, 5.7)	*0.01*
Readmission		
No	Ref	*–*
Yes	0.7 (−1.1, 2.6)	*0.44*
Cervical x CIC	0.1 (−5.9, 6.2)	*0.96*
Cervical x IUC/CIC	10.7 (5.3, 16.2)	*<0.001*
Cervical x IUC	2.1 (−2.8, 7.1)	*0.39*

## Discussion

To our knowledge, this is the first study that quantified different bladder management methods in the United States acute hospital setting for adults with SCI [17]. Nearly half of the sample did not require a catheter during their admission, which may be attributed to the heterogenous population at this level of care and larger sample size. Bladder management was significantly associated with length of acute hospital stay, especially for patients who sustained a cervical level injury and were managed using both IUC and CIC. Our results provide helpful information about existing care patterns that can guide future quality improvement initiatives or interventions aimed at bladder management early after SCI.

The most common bladder management method in our sample was voiding without a urinary catheter (49.7%), which is higher relative to SCI populations in an acute inpatient rehabilitation facility [18]. This discrepancy likely reflects differences in the study setting (i.e., acute hospital versus acute inpatient rehabilitation). Patients who are admitted to acute rehabilitation for SCI tend to have a more severe classification of SCI (i.e., cervical level injury or complete injury) and often require more intensive bladder management methods to treat the associated neurogenic bladder. In contrast, our study examined a broader cohort of individuals with SCI who were admitted to the acute hospital which allowed us to capture the full spectrum of SCI patients across varying levels of injury severity and recovery trajectories, including those who may not transition to acute rehabilitation. By examining this diverse population, our study provides a more comprehensive understanding of bladder management practices at the acute hospitalization level. Additionally, the present study was unable to quantify non-catheter interventions that assist with bladder management, such as oral medications and manual maneuvers, within the no catheter group. It also remains unclear to what extent the no catheter group was fully emptying their bladders with each void. Such details would have been helpful in fully understanding the broader picture of bladder management in the population with SCI and remain areas for future research.

Across the four bladder management groups, only 15.7% admissions were in the CIC-only group, making this the least common strategy. In contrast, two studies that examined bladder management at discharge from acute inpatient rehabilitation estimated that prevalence of CIC usage was nearly 40% [18, 19]. Further, an estimated 33% of adults with SCI used CIC for bladder management in the chronic phase of recovery [2, 20]. This discrepancy is likely explained by the challenges of initiating CIC during an acute hospitalization. Initiating CIC during an acute hospitalization is difficult due to fluctuations in medical acuity or transitions in care (e.g., transferring from an intensive care unit to a trauma floor). The use of continuous intravenous fluids to manage spinal shock or other medical conditions often warrants the use of IUC to manage higher urine output volumes [21]. Additionally, the level of SCI injury and associated impairments (e.g., loss of hand function) impacts a patient’s ability to engage in a CIC protocol. Care teams may also lack confidence, knowledge, or bandwidth to initiate regular CIC during the acute hospitalization. Beyond medical acuity and complexity, patients are often adjusting to the implications of their SCI and may not be emotionally or psychologically ready to engage in CIC management.

Up to 63.1% of the combined IUC and CIC group implemented CIC more than once during an admission. This suggests that the transition between IUC and CIC was not straightforward for many patients. A hospital admission where a mixture of bladder management methods is used likely reflects the fluctuating trajectory of illness and recovery. Acute medical problems that arise will necessitate management one way, and recovery allows the medical team to trial a different method when appropriate. Crucially, these times of transition during acute hospitalization may be periods where the bladder is most prone to overdistension. Future work may want to examine how transitions between bladder management strategy may result in an increase in bladder overdistention, autonomic dysreflexia events, and/or urinary tract infections.

Nearly 84.3% of admissions for patients with SCI remained on a singular bladder management method throughout an admission. Medical teams in the acute hospital setting may be disinclined to experiment with bladder management methods among a SCI population. This may be due, in part, to knowledge gaps regarding best practices for patients with SCI. In one case series, care teams sought to transition patients from IUC (abiding by an infection control IUC-related protocol in at least one situation) but inadequately managed bladder outputs afterwards, leading to acute kidney injury and autonomic dysreflexia [22]. The decision of which bladder management method to employ in patients with SCI is complex, and these results suggest opportunities for further collaboration between acute medical teams and physiatrists to help determine which bladder management method is most appropriate for each patient.

### Limitations

There are limitations to consider when interpreting these results. First, a strength of this study was that the sample that was racially and socioeconomically diverse, relative to other cohorts with SCI that have been evaluated [17, 23, 24]. In turn, this may impact the generalizability of our findings to other settings such as smaller, non-academic, or rural hospitals that may have different policies or care patterns than those that were described in this study. Second, we were unable to obtain the American Spinal Injury Association Impairment Scale (AIS) score, degree of trauma, and acuity for this cohort based on available EHR data elements. As a result, the regression analysis was not adjusted for severity of injury or medical complexity, leaving it susceptible to residual confounding. The AIS score, which reflects the level of neurological impairment and completeness of injury, is not routinely evaluated or recorded within the EHR at the acute level in this health system. This limitation restricts our ability to assess how injury severity or completeness influences bladder management methods and hospital length of stay. Additionally, individuals with cervical injuries who were managed using both IUC and CIC may have greater medical acuity or complexity contributing to their longer hospitalizations [16]. We also did not have data reflecting potential adverse outcomes that may have contributed to a longer length of acute hospitalization. Lastly, the variability between medical staff when documenting urine output in the EHR may have impacted the accuracy of classification. To mitigate this risk, we took several steps to ensure the accuracy of our classification (e.g., corroborating urine outputs with active bladder management orders) but were unable to fully eliminate this risk.

## Conclusion

This study quantified bladder management methods for patients with SCI in the acute hospital setting in the United States. Findings from this study provide necessary descriptive data for future quality improvement initiatives to facilitate optimal, evidence-based bladder management for patients with SCI. Examples of these initiatives include clinical decision support tools to help the care team identify the most appropriate bladder management method for each person or providing feedback to the care team on bladder management and patient outcomes. Individuals with SCI often have multiple, complex medical needs, requiring ongoing, collaborative care between medical disciplines such as neurosurgery, urology, and physiatry. Such multidisciplinary collaboration early during a hospital admission may help address hesitancy among the acute care team for initiating certain bladder management protocols in this population and possibly reducing complications during the hospital course.

## Supplementary information

Supplement

## Data Availability

Data are available from the corresponding author upon reasonable request.
