# Supplementary material for: Bladder management for adults with spinal cord injury in the acute hospital setting: A retrospective study
Source: Spinal Cord Ser Cases. 2026 Mar 5;12:4. doi: 10.1038/s41394-026-00730-8 (PMC12963426; doi:10.1038/s41394-026-00730-8)
Supplement: Supplementary file 1 — Supplement [file 41394_2026_730_MOESM1_ESM.docx]

**Table S1. ICD-10 diagnosis codes for SCI definition**

| **ICD-10 diagnosis code** | **Code description** |
| --- | --- |
| G82* | Paraplegia (paraparesis) and quadriplegia (quadriparesis) |
| S12.0 | Fracture of first cervical vertebra |
| S12.2 | Fracture of third cervical vertebra |
| S13.0 | Traumatic rupture of cervical intervertebral disc |
| S13.2 | Dislocation of other and unspecified parts of neck |
| S13.4 | Sprain of ligaments of cervical spine |
| S14.0 | Concussion and edema of cervical spinal cord |
| S14.1 | Other and unspecified injuries of cervical spinal cord |
| S22.0 | Fracture of thoracic vertebra |
| S23.1 | Subluxation and dislocation of thoracic vertebra |
| S24.0 | Concussion and edema of thoracic spinal cord |
| S24.1 | Other and unspecified injuries of thoracic spinal cord |
| S32.0 | Fracture of lumbar vertebra |
| S33.1 | Subluxation and dislocation of lumbar vertebra |
| S34.0 | Concussion and edema of lumbar and sacral spinal cord |
| S34.1 | Other and unspecified injury of lumbar and sacral spinal cord |
| S34.3 | Injury of cauda equina |
| T06.0 | Injuries of brain and cranial nerves with injuries of nerves and spinal cord at neck level |
| T06.1 | Injuries of nerves and spinal cord involving other multiple body regions |
| T09.3 | Injury of spinal cord, level unspecified |
| T91.3 | Sequelae of injury of spinal cord |

Abbreviations: ICD-10, International Classification of Diseases 10th Revision; SCI, spinal cord injury.
